# Fat infiltration of paraspinal muscles as an independent risk for bone nonunion after posterior lumbar interbody fusion

**DOI:** 10.1186/s12891-022-05178-z

**Published:** 2022-03-09

**Authors:** Gengyu Han, Da Zou, Zexiang Liu, Bo Zhang, Chunjie Gong, Siyu Zhou, Wei Li, Zhuoran Sun, Weishi Li

**Affiliations:** 1 Department of Orthopaedics, Peking University Third Hospital, No. 49 NorthGarden Road, Haidian District, Beijing, 100191 China; 2 Engineering Research Center of Bone and Joint Precision Medicine, Ministry of Education, Beijing, China; 3 Beijing Key Laboratory of Spinal Disease Research, Beijing, China

**Keywords:** Bone nonunion, Fat infiltration, Paraspinal muscle, Posterior lumbar interbody fusion

## Abstract

**Background:**

The prognosis value of paraspinal muscle degeneration on clinical outcomes has been revealed. However no study has investigated the effect of the fat infiltration (FI) of paraspinal muscles on bone nonunion after posterior lumbar interbody fusion (PLIF).

**Methods:**

Three hundred fifty-one patients undergoing PLIF for lumbar spinal stenosis with 1-year follow-up were retrospectively identified. Patients were categorized into bone union (*n* = 301) and bone nonunion (*n* = 50) groups based on dynamic X-ray at 1-year follow-up. The relative total cross-sectional area (rTCSA) and FI of multifidus (MF) and erector spinae (ES), and the relative functional CSA (rFCSA) of psoas major (PS) were measured on preoperative magnetic resonance imaging.

**Results:**

The nonunion group had a significantly higher MF FI and a higher ES FI and a smaller MF rTCSA than the union group (*p* = 0.001, 0.038, 0.026, respectively). Binary logistic regression revealed that MF FI (*p* = 0.029, odds ratio [OR] = 1.04), lumbosacral fusion (*p* = 0.026, OR = 2193) and length of fusion (*p* = 0.001, OR = 1.99) were independent factors of bone nonunion. In subgroup analysis, in one or two-level fusion group, the patients with nonunion had a higher MF FI and a higher ES FI than those of the patients with union (all *p* < 0.05). Similarly, in lumbosacral fusion group, the patients with nonunion had a higher MF FI and a higher ES FI than those of the patients with union (all *p* < 0.05). The logistic regressions showed that MF FI remained an independent factor of bone nonunion both in the patients with one or two-level fusion (*p* = 0.003, OR = 1.074) and in the patients with lumbosacral fusion (*p* = 0.006, OR = 1.073).

**Conclusions:**

Higher fatty degeneration was strongly associated with bone nonunion after PLIF. Surgeons should pay attention to the FI of paraspinal muscles when performing posterior surgery for patients, especially those who need short-segment fusion or to extend fusion to S1.

**Supplementary Information:**

The online version contains supplementary material available at 10.1186/s12891-022-05178-z.

## Background

Posterior lumbar interbody fusion (PLIF) is a commonly surgical treatment for degenerative spinal diseases so as to stabilize the motion segment, restore lordosis and correct deformity [1]. However, bone nonunion as a surgical complication can be observed during follow-up. Most recent studies have reported that the rate of bone nonunion ranged from 7 to 20% [1–3]. The process of bone union can be affected by multiple factors including increased fused level, osteoporosis and obesity [4, 5].

The predictive value of paraspinal muscle morphometry on operative complications has been investigated [6–9]. Some studies reported that decreased cross-sectional area (CSA) of paraspinal muscles was correlated to bone nonunion rate in patients with lumbar surgery [3, 7]. However, the effect of paraspinal muscles fat infiltration (FI) on bone nonunion remains indistinct. We hypothesized that the patients with higher FI were inclined to occur bone nonunion. This study aimed to examine the relationship between FI of paraspinal muscles and bone nonunion in patients with lumbar spinal stenosis (LSS) after PLIF.

## Methods

Hospitalized patients undergoing PLIF for LSS between July 2011 and December 2015 were reviewed. Inclusion criteria were: (1) aged ≥50 years, (2) underwent lumbar magnetic resonance imaging (MRI) and lumbar computed tomography (CT) within 3 months before the index surgery, (3) underwent follow-up of ≥12 months. Exclusion criteria were (1) previous spinal surgery, (2) patients with bone tumor, ankylosing spondylitis, diffuse idiopathic skeletal hyperostosis, rheumatoid arthritis, tuberculosis, or secondary osteoporosis, and (3) patients with scoliosis (> 10°).

A total of 351 patients were identified. Among them, 244 patients with PLIF were LSS and 107 were LSS combined with degenerative lumbar spondylolisthesis. Degenerative lumbar spondylolisthesis was defined as displacement of 1 vertebra over subjacent vertebra using Meyerding grading system [10]. All surgical strategies and approaches were discussed and decided before surgery. For PLIF procedures, using the posterior midline approach, meticulous exposure of the spine and posterior decompression fusion and fixation with pedicle screw was performed. After pedicle screws had been implanted, the neural decompression by laminectomy and discectomy was performed. A polyetheretherketone (PEEK) cage packed with autogenous bone was placed into the interbody space for all patients. Posterolateral fusion was also performed simultaneously. The autograft was harvested from decompression. No bone morphogenetic protein has been used in these patients.

### Bone union evaluation

Segmental fusion status was evaluated by dynamic X-ray at 1-year follow-up. We defined the bone nonunion as 1) there was no continued bone fusion mass at any fusion segment; 2) any motion (greater than 3 mm or 3°) on flexion/ extension plain radiographs [11, 12]. Based on dynamic X-ray, patients were categorized into bone union (*n* = 301) and bone nonunion (*n* = 50) groups.

### Bone density evaluation

**Fig. 1:**
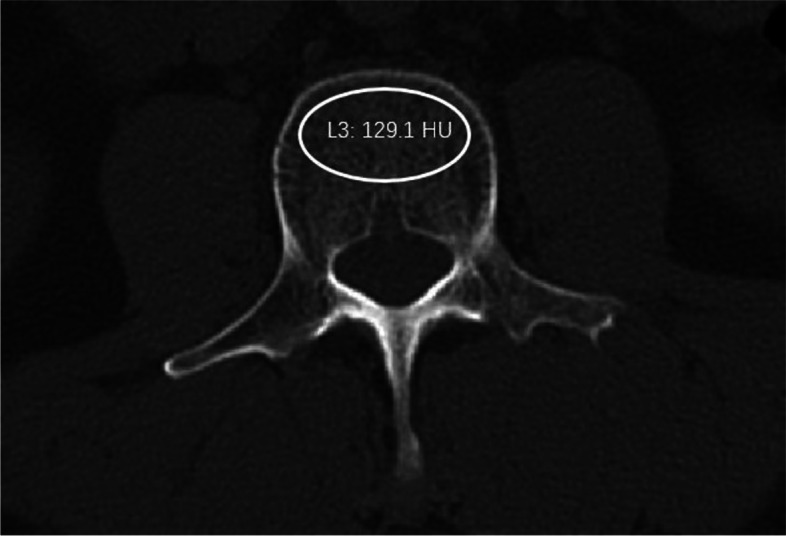
Example of the measurement of HU value: the HU value of L3 was 129.1

In consideration of the overestimation of the BMD of the lumbar spine in patients with lumbar degenerative diseases evaluated by dual-energy X-ray absorptiometry, three-dimensional reconstructive lumbar CT (Siemens, DEFINITION, tube voltage 120 kV) were performed preoperatively to measure the bone density. The Hounsfield unit (HU) value of L1 to L4 was measured for each patient according to the method of previous studies [13]. An oval region of interest inclusive of trabecular bone was placed in the middle-axial CT image of vertebral body (Fig. 1). The cortical bone and posterior venous plexus were excluded in the measurement. The average HU value of L1-4 was calculated.

### Paraspinal muscle evaluation on MRI

**Fig. 2:**
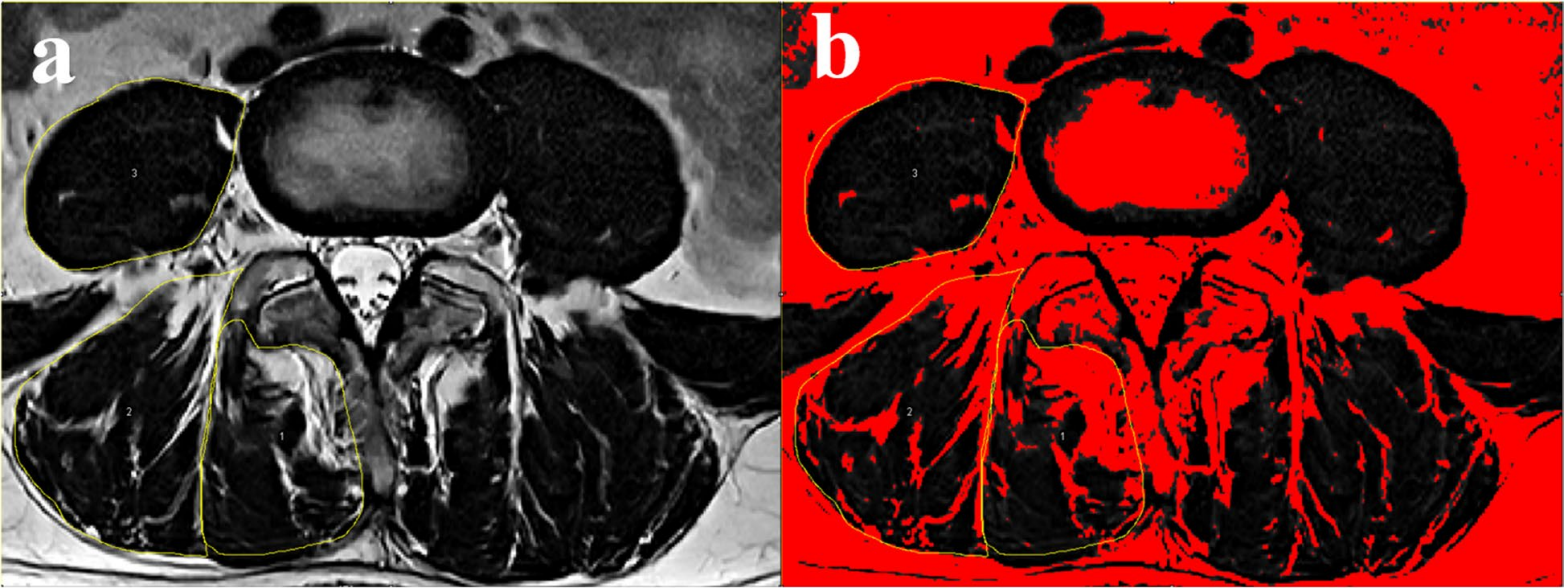
**a** Measurements of paraspinal muscular parameters on axial T2-weighted MRI (a 62-year-old woman). Regions of multifidus (1), erector spinae (2) and psoas muscle (3) at L4 level were outlined by yellow lines. For psoas muscle, only functional muscle was outlined. **b** Thresholding technique to highlight fatty area (red area)

All enrolled patients had undergone preoperative MRI of lumbar area with Signa HDxt 3.0 T (General Electric Company). We measured the multifidus (MF), erector spinae (ES) and psoas major (PS) bilaterally from T2-weighted images at the center of the intervertebral disc of L4-5 level. The following parameters were measured on each level by the Image J software (National Institutes of Health, Bethesda, MD, USA; Fig. 2): total cross-sectional area (TCSA) of MF, ES and intervertebral disc; FI of MF and ES was measured by the previously reported thresholding technique [14, 15]; For PS, only functional cross-sectional area (FCSA) was measured due to the ill-defined outline of intramuscular fat and soft tissue [8]. Thresholding technique can identify two different signal intensity peaks and classify the pixel areas with lower intensity peaks as muscle tissue and the pixel areas with higher intensity peaks as intramuscular fat. Relative cross-sectional area (rCSA, the ratio of cross-sectional area of muscle to that of disc at the same level) was introduced to reduce the effect of body shape on muscular parameters [8, 16]. rCSA of both total muscle (T) and functional muscle (F) were marked as rTCSA and rFCSA.

To test the reliability, all muscular parameters of 10 patients were randomly selected and were measured by two observers independently. After 3 weeks, the same measurements were performed by each observer. The ICCs for both intra-rater and inter-rater reliability of MF rTCSA, ES rTCSA, MF FI, ES FI and PS rFCSA were > 0.8 (Supplement Table 1).

### Statistical analyses

The Mann-Whitney U test or Analysis of Variance (ANOVA) test (for continuous data) and Chi-square test (for categorical data) were conducted to determine the statistical difference of clinical characteristics and paraspinal muscle degeneration between the union group and the nonunion group. Binary logistic regression model was used to identify the independent risk factors of bone nonunion. Age, sex, HU value, smoking, lumbosacral fusion, number of fusion levels, and muscular parameters that had significant differences between groups were included in the regression model. Furthermore, we have performed subgroup analyses according to fusion length and lumbosacral fusion to compare the difference of clinical characteristics and paraspinal muscle between the union and the nonunion groups. Binary logistic regressions were also performed in subgroups. Intraclass correlation coefficient was calculated to test the intra- and inter-rater reliability. Statistical significance was set at *P* value < 0.05. All statistical analyses were performed using SPSS 22.0 (IBM Corp).

## Results

### Demographic data

The bone nonunion occurred in 50 (14.2%) patients. Compared with union group, nonunion group had significantly older age, higher rate of fusions extending to the sacrum, longer fusion length, lower mean HU value of L1-4 and higher rate of smoking (*p* < 0.001, *p* = 0.002, *p* < 0.001, *p* = 0.013, *p* = 0.036, respectively). However, the gender, BMI, whether combined with spondylolisthesis or diabetes were not significantly different between two groups (all *p* > 0.05) (Table 1).

**Table 1:** Descriptive summary of patients between the union group and the nonunion group

	Union Group (*n* = 301)	Nonunion Group (*n* = 50)	*p*-value
Age (year)	60.18 ± 6.55	63.72 ± 7.25	0.001
Sex (male/female)	114/187	23/27	0.275
BMI (kg/cm2)	25.93 ± 3.46	25.89 ± 3.22	0.89
Fusion to S1 (yes)	110/301	30/50	0.002
Number of fusion levels	1.78 ± 0.8	2.44 ± 0.81	< 0.001
Mean HU value of L1-4	128.78 ± 40.53	113.73 ± 35.63	0.013
Spondylolisthesis (yes)	96/301	11/50	0.159
Smoking (yes)	43/301	13/50	0.036
Diabetes (yes)	45/301	8/50	0.848

### Paraspinal muscle characteristics

As shown on Table 2, MF FI and ES FI at L4 were significant higher in nonunion group than in union group (*p* = 0.001, 0.038, respectively). Besides, those who did not achieve union had a smaller MF rTCSA than patients with union (*p* = 0.026). However, there were no significant difference of ES rTCSA and PS rFCSA between two groups (both *p* > 0.05).

**Table 2:** Comparison of paraspinal muscle characteristics between the union group and the nonunion group

	Union Group (*n* = 301)	Nonunion Group (*n* = 50)	*p*-value
L4			
MF FI	0.27 ± 0.12	0.33 ± 0.11	0.001
ES FI	0.21 ± 0.11	0.23 ± 0.08	0.038
MF rTCSA	0.48 ± 0.13	0.46 ± 0.21	0.026
ES rTCSA	0.75 ± 0.2	0.75 ± 0.25	0.5
PS rFCSA	0.58 ± 0.16	0.55 ± 0.15	0.44

### Logistic regression

Furtherly, binary logistic regression revealed that MF FI of L4 was an independent factor of bone nonunion (*p* = 0.029, OR = 1.04; Table 3). Lumbosacral fusion was also an independent factor of bone nonunion (*p* = 0.026, OR = 2.193; Table 3). Besides, the number of fusion levels had a dramatically negative impact on union status (*p* = 0.001, OR = 1.99; Table 3).

**Table 3:** Independent risk factors of bone nonunion identified by logistic regression

	Odds Ratio (95% Confidence Interval)	*P*
Age (+ 1 year)	1.039(0.981,1.1)	0.194
Sex (female)	0.749(0.296,1.896)	0.542
Mean HU value of L1-4	0.991(0.981,1.001)	0.072
Smoking (yes)	2.67(0.989,7.208)	0.053
Lumbosacral fusion (yes)	2.193(1.097,4.385)	0.026
Number of fusion level (+ 1)	1.99(1.342,2.951)	0.001
L4 MF rTCSA (+ 1)	0.791(0.082,7.585)	0.839
L4 MF FI (+ 1%)	1.04(1.004,1.078)	0.029
L4 ES FI (+ 1%)	0.988(0.948,1.03)	0.575

### Subgroup analyses according to fusion length and lumbosacral fusion

In subgroup analysis, the patients were divided into 2 sets of subgroups according to the fusion length and whether lumbosacral fusion was performed respectively. In the patients with one or two-level fusion, the nonunion group had a higher MF FI and a higher ES FI than those of the union group (*p* = 0.001, 0.014, respectively; Table 4). However, there was no difference of muscular parameters between the two groups in the patients with three or more-level fusion (all *p* > 0.05; Table 4). The logistic regression showed that MF FI was still an independent factor of bone nonunion in the patients with one or two-level fusion (*p* = 0.003, OR = 1.074; Supplement Table 2). Additionally, in the patients with lumbosacral fusion, the nonunion group had a higher MF FI and a higher ES FI than those of union group (*p* < 0.001, 0.021, respectively; Table 5), whereas the nonunion group had relatively higher MF FI and ES FI than those of union group without significance (all *p* > 0.05; Table 5). In the patients with lumbosacral fusion, MF FI remained an independent factor of bone nonunion in the logistic regression (*p* = 0.006, OR = 1.073; Supplement Table 3).

**Table 4:** Comparison of paraspinal muscle characteristics between the union group and the nonunion group in subgroup analysis according to fusion length

	One or two-level Fusion Group			Three or More Level Fusion Group		
	Union Group (*n* = 252)	Nonunion Group (*n* = 30)	*p*-value	Union Group (*n* = 49)	Nonunion Group (*n* = 20)	*p*-value
Age (year)	59.65 ± 6.55	62.57 ± 7.61	0.039	62.92 ± 5.92	65.45 ± 6.48	0.213
Sex (male/female)	90/162	12/18	0.644	24/25	11/9	0.65
BMI (kg/cm2)	26.25 ± 3.36	25.79 ± 3.44	0.49	25.34 ± 2.98	26.62 ± 3.53	0.158
Number of fusion levels	1.5 ± 0.5	1.87 ± 0.35	< 0.001	3.22 ± 0.42	3.3 ± 0.47	0.516
Fusion to S1 (yes)	88/252	15/30	0.105	22/49	14/20	0.058
Mean HU value of L1-4	128.41 ± 40.8	114.27 ± 32.3	0.116	130.74 ± 39.47	112.93 ± 41	0.097
Smoking (yes)	34	9	0.028	9	4	0.56
MF FI	0.27 ± 0.11	0.34 ± 0.11	0.001	0.29 ± 0.15	0.3 ± 0.1	0.338
ES FI	0.21 ± 0.1	0.24 ± 0.07(0.23 ± 0.02)	0.014	0.24 ± 0.11	0.22 ± 0.09	0.579
MF rTCSA	0.49 ± 0.14	0.49 ± 0.25	0.206	0.45 ± 0.12	0.41 ± 0.14	0.195
ES rTCSA	0.75 ± 0.19	0.77 ± 0.26	0.773	0.7 ± 0.22	0.72 ± 0.23	0.771
PS rFCSA	0.58 ± 0.16	0.54 ± 0.14	0.261	0.57 ± 0.17	0.56 ± 0.15	0.853

**Table 5:** Comparison of paraspinal muscle characteristics between the union group and the nonunion group in subgroup analysis according to lumbosacral fusion

	Non-lumbosacral fusion Group			Lumbosacral fusion Group		
	Union Group (*n* = 191)	Nonunion Group (*n* = 20)	*p*-value	Union Group (*n* = 110)	Nonunion Group (*n* = 30)	*p*-value
Age (year)	60.93 ± 6.96	62.8 ± 6.61	0.208	58.88 ± 5.71	64.33 ± 7.69	< 0.001
Sex (male/female)	79/112	11/9	0.241	35/75	12/18	0.4
BMI (kg/cm2)	25.98 ± 3.51	25.98 ± 3.86	0.998	25.84 ± 3.39	25.82 ± 2.8	0.985
Number of fusion levels	1.63 ± 0.75	2.1 ± 0.72	0.008	2.04 ± 0.83	2.67 ± 0.8	< 0.001
Mean HU value of L1-4	127.09 ± 38.78	120.65 ± 43.8	0.386	131.73 ± 43.42	109.12 ± 28.88	0.018
Smoking (yes)	30	8	0.013	13	5	0.333
MF FI	0.28 ± 0.12	0.29 ± 0.07	0.335	0.26 ± 0.1(0.27 ± 0.1)	0.35 ± 0.12(0.35 ± 0.2)	< 0.001
ES FI	0.21 ± 0.11	0.22 ± 0.07	0.475	0.2 ± 0.1(0.21 ± 0.01)	0.24 ± 0.08(0.23 ± 0.02)	0.021
MF rTCSA	0.5 ± 0.14	0.44 ± 0.1	0.066	0.46 ± 0.12	0.47 ± 0.26	0.399
ES rTCSA	0.75 ± 0.2	0.72 ± 0.22	0.33	0.74 ± 0.19	0.77 ± 0.27	0.984
PS rFCSA	0.59 ± 0.17	0.56 ± 0.17	0.496	0.55 ± 0.15	0.54 ± 0.13	0.98

## Discussion

Our study showed that the patients who did not achieve union had a higher MF FI and a higher ES FI than those of the patients with union. In Lee et al’s study, it was reported that the union rate decreased as fat content of extensor muscles increased, which was accordant to our findings [17]. However, they only used a semiquantitative scale to quantify the FI and did not investigate the MF and ES separately. Considering that MF is in the deep attaching to the lumbar vertebrae while ES is more superficial spanning more sections of the spine, evaluating them separately is reasonable [18]. Our results were also consistent to Katsu et al’s study that focused on the patients with osteoporotic fractures [19]. They found that FI of MF and ES were both higher in insufficient union group than in union group. Several studies have revealed that increased muscle FI was correlated to poorer muscle strength [20, 21]. Previous study indicated that incremental bending moment transmitted by the internal fixation device would increase the risk of bone nonunion [22]. Consequently, paraspinal muscles with higher FI might be less effective on reducing the bending moment. Besides, desired paraspinal musculature could provide important vascular ingrowth into the fusion site and accelerate the bone healing [23]. It is suggested that severe muscular degeneration might impede this process.

Of note, multifactor analysis demonstrated that FI of MF rather than ES, had an effect on nonunion. Liu et al. investigated 118 LSS patients and found that the postoperative improvement of Oswestry Disability Index (ODI) was significantly less in MF FI ≥ 25% group than in MF FI < 25% group [24]. Besides, Hong et al. found that MF FI also contributed to superior clinical outcomes including less improvement in ODI, greater postoperative pain and higher reoperation rate [25]. As MF is the innermost and largest one of the paraspinal muscles and provides two-thirds of spinal segmental stability [26], MF might have a more remarkable effect on clinical outcomes compared to ES.

Our analysis showed that the nonunion group had a greater MF atrophy than that of union group, but no statistical significance was seen in multivariate analysis. Choi et al’s study reported that CSA of MF, ES and PS in the nonunion group were all smaller than those in the union group [7]. Furthermore, they found that only PS TCSA was correlated to fusion rate in multivariate analysis. The reason why the TCSA did not show an arresting effect on bone nonunion might be that the relationship between TCSA and muscle strength was not as significant as that of FI. A study demonstrated that FI of paraspinal muscles, not CSA, remained a significant predictor of extensor strength in multivariate regression [20]. Our study indicated that surgeons should pay more attention to the FI of paraspinal muscles rather than atrophy when evaluating the risk of bone nonunion preoperatively.

Multivariable analysis showed that the number of levels fused significantly affected the incidence of nonunion. In previous studies, the number of fusion levels was considered to be a crucial factor in achieving solid fusion in degenerative lumbar diseases [17, 22]. In view of the possible correlation between fusion length and the degree of preoperative FI, we performed a subgroup analysis. The results exhibited that the effect of FI only existed in the patients with one or two fused levels, not in the patients with three or more fused levels. We speculated that in the patients with long-segment fusion, the fusion length had a more notable effect on the bone nonunion over paraspinal muscles FI, hence a severe fatty degeneration might not notably increase the risk of nonunion. Nevertheless, in the patients with shorter fused levels, FI of paraspinal muscles began to take effect.

In addition, we found that the patients with fusion to S1 had a higher rate of bone nonunion, which was consistent with previous studies [17, 22, 27]. In subgroup analysis, the significant difference of FI between the union group and the nonunion group only existed in the patients with lumbosacral fusion, not in the patients without lumbosacral fusion. It could be interpreted by that as a great mechanical load could be applied to the sacrum in lumbosacral fusion, patients need stronger paraspinal muscles to counteract this negative effect [17]. Once patients have a higher FI of muscles preoperatively, lumbosacral fusion will highlight the effect of muscles and then the risk of bone nonunion will increase.

Our findings indicated that surgeons should pay attention to the FI of paraspinal muscles when performing posterior surgery for patients who need short-segment fusion or to extend fusion to S1. In the above cases, preventive measures such as the use of materials to facilitate bone grafting or screws with greater fixation strength should be considered.

We recognize some limitations in the study. First, there were no postoperative MRIs to evaluate the condition of muscle injury during the operation, which might reduce the predictive value of paraspinal muscles on bone nonunion. Besides, we did not perform CT to evaluate the fusion status which may reduce the reliability of bone nonunion. While in our study, 2 observers evaluated union independently with strict criteria for defining nonunion in order to increase the accuracy. Third, the heterogeneity of cases and the small number of cases for subgroup analysis might produce bias. In addition, we have not taken the size and length of the screw into account, which might be related to bone healing [28]. Moreover, it may be impractical to actually measure FI in clinical practice since automated measurement software is not yet available.

## Conclusions

This is the first study focusing on the prognostic value of back muscles FI to predict bone nonunion after PLIF. We demonstrated that higher fatty degeneration of MF was an independent factor of nonunion. Furtherly, the effect of FI only existed in the patients with one or two fused levels, not in the patients with three or more fused levels. Besides, the significant difference of FI between the union group and nonunion groups only existed in the patients with lumbosacral fusion, not in the patients without lumbosacral fusion. In cases with higher MF FI during preoperative evaluation, we considered that more rigid fixation or more graft bone might be necessary.

## Supplementary Information

**Additional file 1: Supplement Table 1.** Intra-rater and inter-rater reliability of paraspinal muscle parameters using intraclass correlation coefficient. **Supplement Table 2**. Independent risk factors of bone nonunion identified by logistic regression in the one or two-level fusion group. **Supplement Table 3**. Independent risk factors of bone nonunion identified by logistic regression in the lumbosacral fusion group.

## Data Availability

The datasets used and/or analyzed during the current study are available from the corresponding author on reasonable request.

## Abbreviations

CSA: Cross-sectional area
CT: Computed tomography
ES: Erector spinae
FCSA: Functional cross-sectional area
FI: Fat infiltration
HU: Hounsfield unit
LSS: Lumbar spinal stenosis
MF: multifidus
MRI: Magnetic resonance imaging
ODI: Oswestry Disability Index
PEEK: Polyetheretherketone
PLIF: Posterior lumbar interbody fusion
PS: Psoas major
rCSA: Relative cross-sectional area
TCSA: Total cross-sectional area
