# Supplementary material for: Fat infiltration of paraspinal muscles as an independent risk for bone nonunion after posterior lumbar interbody fusion
Source: BMC Musculoskelet Disord. 2022 Mar 9;23:232. doi: 10.1186/s12891-022-05178-z (PMC8908625; doi:10.1186/s12891-022-05178-z)
Supplement: Supplementary file 1 — Additional file 1: Supplement Table 1. Intra-rater and inter-rater reliability of paraspinal muscle parameters using intraclass correlation coefficient. Supplement Table 2. Independent risk factors of bone nonunion identified by logistic regression in the one or two-level fusion group. Supplement Table 3. Independent risk factors of bone nonunion identified by logistic regression in the lumbosacral fusion group. [file 12891_2022_5178_MOESM1_ESM.docx]

Supplement table 1. Intra-rater and inter-rater reliability of paraspinal muscle parameters using intraclass correlation coefficient.

|  | Intra-rater | inter-rater |
| --- | --- | --- |
| MF FI | 0.86 | 0.833 |
| ES FI | 0.881 | 0.82 |
| MF rTCSA | 0.938 | 0.922 |
| ES rTCSA | 0.921 | 0.906 |
| PS rFCSA | 0.855 | 0.885 |

Supplement table 2. Independent risk factors of bone nonunion identified by logistic regression in the one or two-level fusion group.

|  | Odds Ratio (95% Confidence Interval) | P |
| --- | --- | --- |
| Age (+1 year) | 1.019(0.951,1.092) | 0.596 |
| Number of fusion level (+1) | 7.28(2.231,23.755) | 0.001 |
| Lumbosacral fusion (yes) | 1.525(0.626,3.712) | 0.353 |
| Smoking (yes) | 0.998(0.985,1.01) | 0.706 |
| Mean HU value of L1-4 | 5.411(1.928,15.183) | 0.001 |
| L4 MF FI (+1%) | 1.074(1.025,1.126) | 0.003 |
| L4 ES FI (+1%) | 0.981(0.93,1.036) | 0.497 |

Supplement table 3. Independent risk factors of bone nonunion identified by logistic regression in the lumbosacral fusion group.

|  | Odds Ratio (95% Confidence Interval) | P |
| --- | --- | --- |
| Age (+1 year) | 1.06(0.978,1.149) | 0.154 |
| Number of fusion level (+1) | 2.246(1.26,4.004) | 0.006 |
| Smoking (yes) | 0.99(0.975,1.005) | 0.187 |
| Mean HU value of L1-4 | 2.338(0.642,8.515) | 0.198 |
| L4 MF FI (+1%) | 1.073(1.021,1.128) | 0.006 |
| L4 ES FI (+1%) | 0.98(0.926,1.037) | 0.48 |
